# Thermoplasmonic Nanorings via Sputter Deposition

**DOI:** 10.3390/ma18184371

**Published:** 2025-09-18

**Authors:** Xavier Baami González, Peter K. Petrov, Duncan S. Sutherland

**Affiliations:** 1 Interdisciplinary Nanoscience Center (iNANO), Aarhus University, Gustav Wieds Vej 14, 8000 Aarhus, Denmark; xbaami@inano.au.dk; 2 Department of Materials, Imperial College London, Prince Consort Rd 8, South Kensington, London SW7 2AZ, UK; p.petrov@imperial.ac.uk

**Keywords:** nanotechnology, plasmonics, nanofabrication, nanorings, DC sputtering

## Abstract

The fabrication of plasmonic nanostructures with precise geometries and scalable production remains a critical challenge for advancing light–matter interaction technologies in applications such as sensing, photonics, and thermal management. Here, we present a versatile, self-assembly-based strategy for metallic nanoring fabrication. We extend Hole-mask Colloidal Lithography (HCL) by employing ring-shaped holes to produce nanorings via direct current (DC) magnetron sputtering. The process relies entirely on industry-standard thin-film techniques, enabling wafer-scale integration. Using this approach, we fabricate copper (Cu) nanorings with tunable near-infrared (NIR) resonances suitable for thermoplasmonic applications. The thermoplasmonic performance of these nanorings is evaluated under direct sunlight, revealing efficient photon-to-heat conversion. Nanorings displayed enhanced heating, outperforming nanodisks of equivalent size, with maximum surface temperatures reaching approximately 37 °C, an increase of over 13 °C above ambient, in contrast to the 6 °C increase shown by disks that reached a temperature of 30 °C. This superior performance is attributed to the nanoring geometry, which promotes stronger light absorption and localized heating. Overall, our results demonstrate that Cu nanorings represent a robust and scalable plasmonic platform with significant potential for solar-driven technologies and thermal management applications.

## 1. Introduction

The increasing demand for advanced materials capable of controlling light–matter interactions at the nanoscale has spurred intensive research into engineered nanostructures [1]. Among these, metallic nanostructures have demonstrated unparalleled capacity for manipulating electromagnetic radiation, particularly in the near-infrared (NIR) and visible regions [2,3]. Such capabilities are central to applications ranging from thermal management and energy-efficient coatings to biochemical sensing and photonic circuitry [4,5]. In this context, nanoring structures emerge as particularly attractive platforms due to their geometry-enabled tunability, compact footprint, and strong plasmonic behavior [6,7,8]. Nanorings are interesting in different fields, including optical sensors [9], surface-enhanced Raman spectroscopy [10,11,12], and biosensing [8]. However, for these nanostructures to transition from laboratory research to practical deployment, the fabrication methodologies must not only deliver high precision and reproducibility, but also meet scalability, cost-effectiveness, and compatibility criteria aligned with industrial manufacturing standards.

Traditional lithographic methods, such as electron beam lithography (EBL) and focused ion beam (FIB) lithography, provide precise control over nanostructure geometries and arrangements, allowing the fabrication of complex and highly tailored nanoscale features [13,14]. However, these methods are intrinsically limited by their serial nature, resulting in high production costs and limited throughput—factors that hinder their direct application to industrial-scale manufacturing [15,16,17]. While photolithography (PL) is widely utilized in industry due to its scalability and parallel processing capabilities, it typically requires substantial initial capital investments and sophisticated infrastructure, rendering it economically impractical for many specialized or low-volume applications [17].

In contrast, self-assembly techniques such as colloidal lithography (CL) offer a low-cost, scalable, and versatile route for nanostructure fabrication [18,19]. These methods typically involve the use of self-assembled monolayers or multilayers of spherical colloidal particles, employed either as deposition masks or templates [20]. A well-known variant is nanosphere lithography (NSL), which uses hexagonally close-packed colloidal arrays as evaporation masks [21]. A more flexible alternative is Hole-mask Colloidal Lithography (HCL) [22,23], which combines a polymer lift-off layer with Sparse Colloidal Lithography (SCL) [24]. Unlike NSL, HCL employs short-range ordered arrays of charged colloids, leading to disordered yet uniformly spaced nanostructures that avoid near-field coupling and long-range diffractive effects—thereby enabling more isolated and reproducible optical responses [22].

Numerous strategies have been explored for the fabrication of nanorings, including several developed within our research group [25,26]. Many of these methods rely on high-cost top-down techniques such as EBL [10], sputtering re-deposition [9,27], soft lithography [28], capillary lithography [29], and even large-scale facilities employing X-ray lithography [30]. While these approaches offer precise morphological control, they are not scalable and involve considerable time and cost investments [31,32,33]. Furthermore, earlier attempts at using self-assembly-based methods for nanorings fabrication often lacked precise control over key parameters such as wall thickness and reproducibility, and were typically limited to gold-based nanostructures [25].

In our previous work, nanoring structures were fabricated using e-beam physical vapor deposition (PVD) [26], which, although suitable for prototyping, presents significant limitations for industrial compatibility due to its low deposition rates, high equipment costs, and restricted material choices. This approach was not compatible with sputter deposition, severely limiting its scalability. To address these constraints, we propose an enhanced version of HCL allowing the use of direct current (DC) sputtering and opening for scalable production of nanorings. This adaptation not only retains the scalability and pattern versatility of colloidal lithography [34] but also leverages the inherent advantages of DC sputtering: uniform material deposition over large areas, compatibility with a wide range of materials, and alignment with industry-standard thin-film processes [35].

By implementing DC sputtering instead of e-beam PVD, the proposed method provides a more robust, time-efficient, and scalable approach to nanoring fabrication [36]. It enables systematic control over ring diameter, while also supporting the use of diverse metallic and dielectric materials. The conformal and homogeneous nature of sputtering deposition ensures reproducibility across large areas, making it highly suitable for wafer-scale production. Additionally, the equipment and process parameters involved in DC sputtering are widely adopted in industrial environments, particularly in the fields of microelectronics and photovoltaics, thereby facilitating integration into the existing manufacturing workflows [37,38,39].

Moreover, this approach surpasses previous solutions, which, although promising on a laboratory scale, entailed significant upfront costs associated with mask production and lacked the material flexibility offered by sputtering techniques [36,39]. In contrast, the current method provides a pathway toward low-cost, high-throughput fabrication of nanoring architectures without compromising structural integrity or optical performance.

## 2. Materials and Methods

### 2.1. Materials and Reagents

Raw materials and reagents were used to prepare the required samples and develop the different tests as received. Silicon wafers (ConScience—Gothenburg, Sweden), glass substrates, size No. 1.5 (VWR), ethanol 96% (EtOH, Technic France—Noyarey, France), acetone (Technic France—Noyarey, France), isopropanol (Technic France—Noyarey, France), dimethyl sulfoxide (Technic France—Noyarey, France), Polydimethyl Glutarimide (PMGI 2S SF, Kayakli—Waltham, MA, USA), Polyethyleneimine (PEI, Sigma—USA-Burlington, MA, USA), polystyrene sulfonate (PSS, Sigma—Burlington, MA, USA), Poly(diallydimethylammonium) (PDDA, Sigma—USA, Burlington, MA, USA), sulfate-modified polystyrene (PS) nanobeads (200, 300, 500, 800, ThermoFisher—USA—Waltham, MA, USA), Titanium (Ti), Gold (Au), silver (Ag), copper (Cu), and platinum (Pt), for magnetron sputtering (Mantis™—London, UK), Nitto Deko SWT10+ Tape (Nitto Scandinavia AB—Lindome, Sweden).

### 2.2. Fabrication of Nanorings—Spin Coating Step

Silicon or glass substrates were cleaned on an ultrasonic bath in acetone for 5 min, rinsed with ethanol or isopropanol, and dried with a N_2_ gas flow. This was followed by submitting the substrates to a reactive ion etching process with O_2_ plasma (3 min. 50 W, 100 mTorr, 100 SCCM O_2_) to remove any residues that were persistent on the surface.

Once the substrates were cleaned, a 75 nm PMGI layer was spin-coated onto the substrates by first depositing a layer of PMGI 2S SF solution onto the surface and then spinning the sample in the corresponding spin coating machine (APV Arias Wet Bench – Specific Design) for 60 s (1000 rpm/s, 500 rpm/s acceleration speed). The thin film was cured by hot baking at 200 °C for 3 min; when completed, the substrates were dried using a N_2_ gas flow.

### 2.3. Fabrication of Nanorings—Colloidal Step

The PMGI spin-coated samples were submitted to reactive ion etching with O_2_ plasma (15 s, 50 W, 25 mTorr, 100 SCCM O_2_) to turn the surface hydrophilic before the polyelectrolyte’s deposition.

Three polyelectrolyte layers were deposited on top of the PMGI thin film: First, a 2 wt% PEI solution was deposited onto the sample (sit for at least 30 s, rinsed with water for 30 s, and dried with an N_2_ gas flow). This was repeated with a 2 wt% PSS solution; the last of the polyelectrolytes was deposited using a PDDA solution (0.1–0.5 wt%, let sit for at least 1 min, rinsed for 1 min, and dried with a N_2_ gas flow).

After depositing the three polyelectrolytes, a solution of 0.2 wt% polystyrene nanoparticles (PS NPs) was deposited, let sit for a range of time depending on the NP size (5–30 min), rinsed for 1 min, placed into boiling water for 1 min, and finally dried with a N_2_ gas flow.

### 2.4. Fabrication of Nanorings—Metal Deposition Step

The colloidal layer was used to create the metallic nanorings by means of direct current (DC) magnetron Sputtering. First, a mask layer of Ti was deposited (15 nm Ti, 0.2 nm/s, 200 W, 8 × 10^−3^ mbar). This was followed by submitting the substrates to reactive ion etching with O_2_ plasma (100 W, 6–20 min, 50 mTorr, 100 SCMM O_2_) to create a hole mask.

After the deposition of the metals, the PS NPs were removed from the surface by tape stripping. Nitto Deko SWT10+ tape was placed on top of the substates’ surfaces, then it was carefully peeled off to remove the tape and bring with it the PS NPs, generating the ring-shaped hole mask.

With the generated mask to fabricate the rings of interest, Cu was deposited on the same system (25 nm Cu, 1 nm/s, 200 W, 3.4 × 10^−3^ mbar).

The excess metal was then removed by liftoff of the PMGI layer, and the substrates were placed in an ultrasonic bath with warm DMSO until the metal layer was no longer visually detected. The last step of the nanorings fabrication was the rinsing with ethanol or isopropanol of the substrates, followed by water and drying with a N_2_ gas flow.

### 2.5. Morphological and Optical Properties Characterization

The fabricated nanorings’ morphology was characterized by imaging the surface of the glass and silicon substrates using both Scanning Electron Microscopy (SEM) and Atomic Force Microscopy (AFM). On the other hand, the optical response was examined using UV-Vis-NIR spectrophotometry.

For SEM imaging, an FEI Magellan 400 SEM machine was used to image the samples. To reduce charging, all the glass samples had 10 nm of Ti deposited before SEM imaging. The samples were imaged using a beam current of 50 pA and an accelerating voltage of 5.00 kV.

For AFM imaging, a Bruker Dimension Edge AFM was used to image the samples. The samples were imaged using tapping mode, using an RTESP-300 tip from Bruker as the imaging tip. Each image was taken by measuring 256 lines in the y-direction, with 256 samples per line, creating a 256 × 256 pixel image, which was later corrected in post-processing if needed.

The optical spectra to examine the optical response of the fabricated nanorings were recorded using a Shimadzu 300 UV-Vis-NIR spectrophotometer. A double-beam setup was used to measure the extinction spectra, where one beam is shot through a cover glass without any nanostructures as a control, while the other beam is shot through a sample containing the nanostructures of interest on a glass substrate. The samples were placed so that the side with the deposited metal nanoring was facing toward the direction of the light, allowing the beam to hit the nanostructures before the substrate itself.

### 2.6. Simulation of Nanorings’ Optical Response

The program Lumerical FDTD Solutions by Ansys (2023 R1) was used to simulate the electromagnetic response of metallic nanostructures on a substrate when subject to light. A simulation space was set up as in Figure S10, with the space surrounded by perfectly matched layers (PML). The specific simulation space parameters used for the simulations can be found in Table S2. Within the simulation space, a glass substrate (simulated as a dielectric with refractive index of 1.52) was placed, with a metallic nanoring on top of the substrate surface. An optical source was placed so that its space surrounds the metal nanoring, while monitors for scattering, absorption, and the e-field response were placed relative to the metal nanoring and the optical source. The selected source emitted a light pulse with a wavelength range of 400–2500 nm.

The nanoring’s dimensions are described by a halved toroid, with a major outer radius (R), a horizontal inner radius (r_a_), and a vertical inner radius (r_b_) in order to simulate the real structures of the fabricated nanorings, see Figure S11. In Table S3, the values of the nanoring parameters for simulating 200, 300, 500, and 800 nm diameters are described.

## 3. Results and Discussion

### 3.1. Overview of the Fabrication Strategy and Evaluation of Replicability

**Figure 1:**
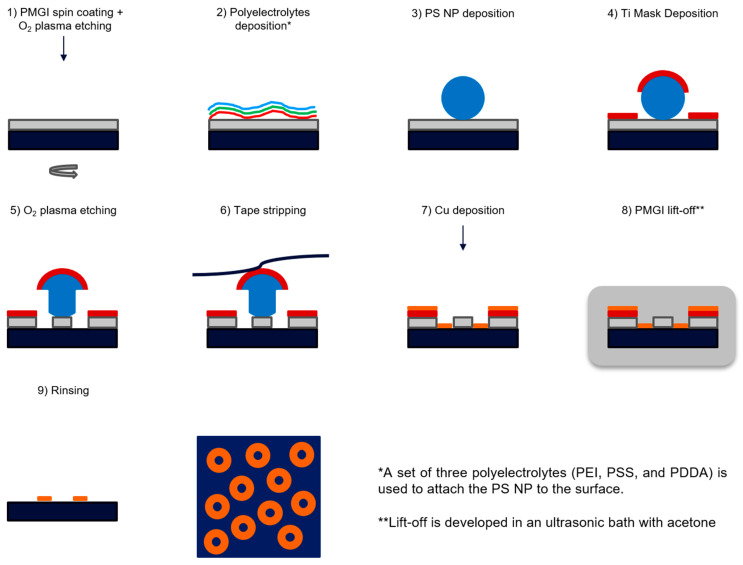
Diagram of the steps in the new fabrication of Cu nanorings combining Hole-mask Colloidal Lithography with DC magnetron sputtering.

We aimed to develop a large-area nanofabrication route that allows the fabrication of nanoscale rings of different materials employing DC magnetron sputtering to match industrial-level standards. Following a similar rationale to our previous work [26], HCL was selected as a technique seeking to benefit from not needing an EBL-defined master. HCL has been well-defined by different authors [22,23]; originally, it was used to fabricate circular nanostructures by PVD through the hole mask and lift-off. In our previous work [26], we modified the process to replace circular apertures in the hole mask with ring-shaped apertures to allow the fabrication of nanoring structures of a range of different materials; this was achieved by maintaining the particles in place during the etching process. However, the work presented required angled deposition of the metal of interest to obtain the final nanostructures. This presents clear geometrical limitations if large surfaces (>500 cm^2^) need to be fabricated. To avoid angled deposition, an alternative method was designed. A schematic of this is shown in Figure 1. The first five steps were taken from our previous fabrication route. The key difference was that the nanoparticle was removed (step 6) before the final deposition step (Step 7), removing the requirement for angled deposition. To maintain nanoring structures, the polymer below the nanoparticle must remain in place.

The design of the new method involved optimization of several parameters, including the thickness of the PMGI layer; the O_2_ plasma etching parameters, e.g., time and power; and the amount of metal to be deposited on the final structures. As shown in our previous work [26], the thickness of the polymer layer is a critical parameter requiring thinner films than traditional HCL, as the O_2_ plasma etching step needs to etch completely through to the substrate before prolonged etching removes the nanoparticle by underetching the polymer beneath it. To study that, a series of experiments was carried out to optimize the thickness of the spin-coating step polymer film. PMGI films of 75 nm thickness were prepared by spin coating after optimization (confirmed by profilometry, e.g., Figure S1). This thickness of PMGI corresponds to the recommended ≥33% thickness margin relative to the deposited metal (~56 nm), which is essential to achieve a clean lift-off and prevent structural defects in the nanorings according to the manufacturer: Kayaku Advanced Materials.

**Figure 2:**
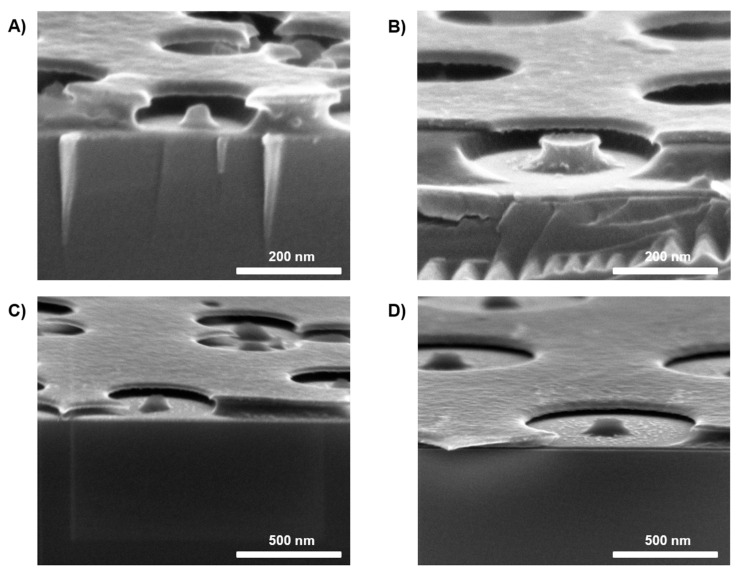
SEM images showing the PMGI pillars when using different sizes of PS NPs: (**A**) 200 nm; (**B**) 300 nm; (**C**) 500 nm; (**D**) 800 nm.

**Figure 3:**
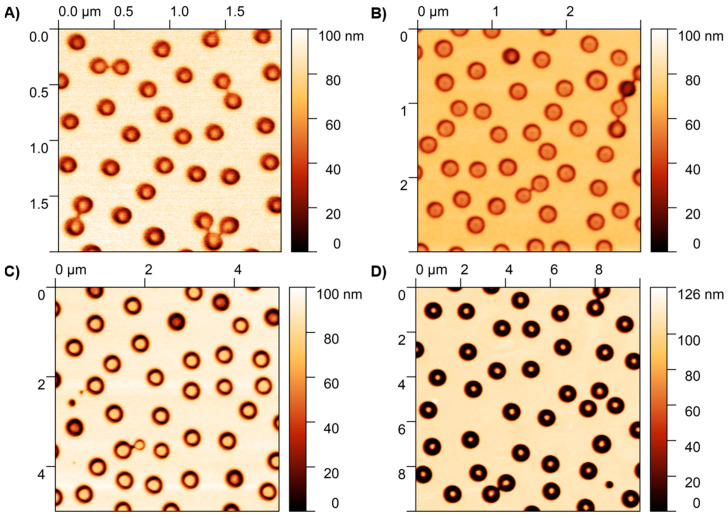
AFM images showing the PMGI pillars when using different sizes of PS NPs: (**A**) 200 nm; (**B**) 300 nm; (**C**) 500 nm; (**D**) 800 nm.

In order to allow the nanoparticle to be removed from the sample without removing the polymer underneath, the adhesion between the the polymer and the substrate needed to be optimized. A new resist was chosen in the present method compared to the previous one; PMGI was introduced instead of PMMA, and the main reason behind this decision was the difference in the adhesion of the resist to the substrate. When using PMMA, after removing the polystyrene nanoparticles (PS NPs) by tape stripping, all the polymer underneath the particles was also removed. We believe that this is due to a stronger interaction between PMMA and the particle than the one between the resist and the substrate. Consequently, this led to circular holes as traditional HCL does, and not to ring-shaped holes. By introducing PMGI as the new resist, we ensured a greater interaction between the polymer and the substrate, and this allowed us to tape strip the PS NPs (Step 6) and, as a result, obtain a pattern of PMGI pillars underneath the mask layer (Figure 2 and Figure 3). This tape-stripping step, leading to the polymer pillars, is key and directly affects the final structures; this is discussed later in this section.

The spin coating of PMGI was followed by SCL to deposit the dispersed colloidal monolayer. The process is well described in our previous work [26]; diverse sizes of PS NPs were used from 200 to 800 nm, obtaining a random sequential adsorption of the beads, leading to short-range ordered arrays (Figure S2). In that work, a deeper analysis of this short-range order can be found; however, it is important to remember that this is desired in order to avoid plasmonic coupling and other effects from the final structures.

A 15 nm mask layer of Ti was then deposited by DC magnetron sputtering (0.2 nm/s, 200 W, 8 × 10^−3^ mbar) to protect the PS NPs during the etching step and define the final pattern of the fabrication of nanorings. This was followed by etching the unprotected PMGI underneath the particles with O_2_ plasma (Figure S3) to generate the ring-shaped cavities for the later metal deposition. Reactive ion etching (RIE) encompasses three primary processes: chemical etching, ion-enhanced chemical etching, and physical sputtering [40]. As we discussed in some of our previous works in the group, ion-assisted chemical etching seems to be the predominant mechanism of etching of the PMGI [26]. In this case, the etch was particularly faster because PMGI is an oxygen-containing polymer, which makes it suitable for a faster etching rate. Moreover, chemical etching leads to the etching of the PS NPs and the underetching of PMGI beneath the Ti mask layer. Among others, process pressure, O_2_ flow rate, power, and time are key parameters that define the quality of the etching and, consequently, the final structures’ morphology and optical response. For the present work, the first three parameters were fixed to 50 mTorr, 100 SCCM O_2_, and 100 W, while the etching time was shifted depending on the size of the particles. Longer process times were needed when using larger particles (Table S1), ranging from 6 (for 200 nm) to 20 min (for 800 nm). We also found that for a specific size, with longer etching treatments, the diameter of the obtained pillars defining the ring-shaped holes was reduced, leading to final nanoring structures with smaller inner diameters and different optical responses [26].

After the etching of the PMGI, the nanoparticles were removed by tape stripping to expose the pillars underneath that worked together with the hole mask as the pattern for the final metallic nanostructures (Figures S4 and S5); these pillars needed to be sufficiently tall (taller than the height of the final structures) to be accessible for the final lift off. As can be seen in Figure 2, these were not completely straight, and this could lead to some aggressive lift-off, causing damage to the final nanostructures; however, the rings had good optical properties.

Despite the pillars not having very vertical sidewalls, their height was very consistent and in accordance with the original thickness of the main layer of the polymer; as shown in the AFM results in Figure 3, most of the polymeric structures maintained the original height. In some cases, the pillars presented shorter height values, which led to a small fraction of encapsulated pillars; these, however, were not observed to alter the overall optical spectra substantially.

Once the PS NPs were tape stripped, Cu was deposited using DC magnetron sputtering to create the nanorings inside the ring-shaped cavities. This was deposited at a rate of 1 nm/s up to an amount of 25 nm (200 W, 3.4 × 10^−3^ mbar). In this case, an adhesion layer of Ti was not needed; however, this was necessary in the previously reported method that used E-beam PVD [26]. This is because DC sputtering provides enough energetic bombardment and dense film growth to naturally promote adhesion, eliminating the need for a Ti adhesion layer in many cases [35].

To reveal the final structures, the substrates were then immersed in an ultrasonic bath with warm DMSO to achieve the lift-off PMGI. This allowed the removal of all the extra material deposited on top of the polymer layer (check Figure S6, which shows images from the different steps). This was concluded by cleaning the substrates with IPA to remove any organic traces and then with deionized water.

**Figure 4:**
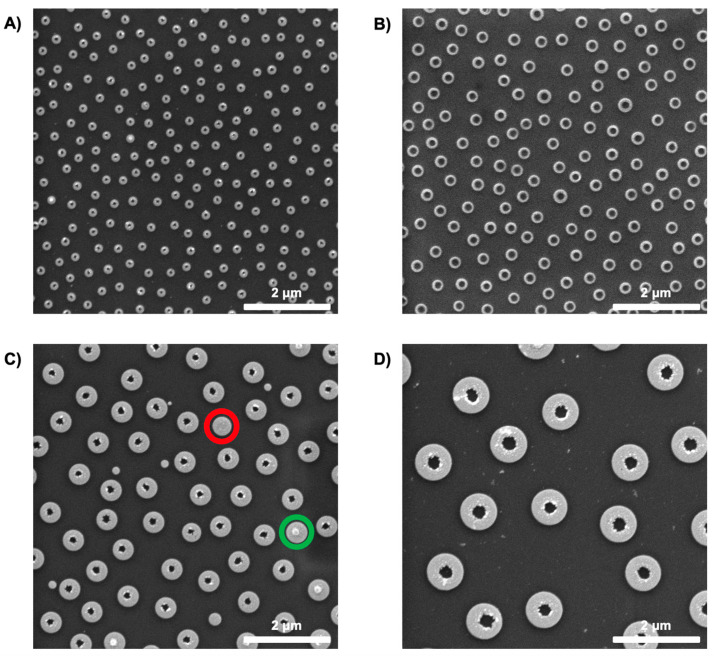
SEM images showing Cu nanorings of different sizes: (**A**) 200 nm; (**B**) 300 nm; (**C**) 500 nm; (**D**) 800 nm.

As seen in the SEM images in Figure 4, arrays of Cu nanorings were obtained after lifting off the PMGI; the images revealed some of the different sizes of structures that can be fabricated by the method. A careful analysis of the nanoring structures reveals that the structures of 200, 500, and 800 nm have a much smaller inner diameter in comparison to that of 300 nm. The diameter of the polymer pillars is a key parameter that determines the final structures. A larger diameter for the pillars led to a larger inner diameter. As observed in Figure 2B, for 300 nm, the pillars present a larger diameter in relation to the overall ring-shaped hole size compared to the other sizes; this leads to rings with an overall larger inner diameter (Figure 3B). Furthermore, the consistent reduction in inner diameter arises from the finite angular spread of the sputtered flux and the slightly sloped PMGI sidewalls, which promote sidewall coating and effectively narrow the aperture beyond the nominal film thickness.

Moreover, as mentioned before, the fact that the pillars’ sidewalls were not fully vertical could cause some problems in the final shape of the structures during the lift-off. In Figure 4C,D, we can see how the inner walls of the nanoring structures present some non-continuous traces; this can be easily explained by an aggressive lift-off caused by non-straight pillars. Another fact that is observed in Figure 4C is the deformity caused by the presence of pillars that do not present the full height, leading to encapsulated pillars (green circle), or when pillars are completely etched or removed with the particles, leading to nanodisks (red circle).

**Figure 5:**
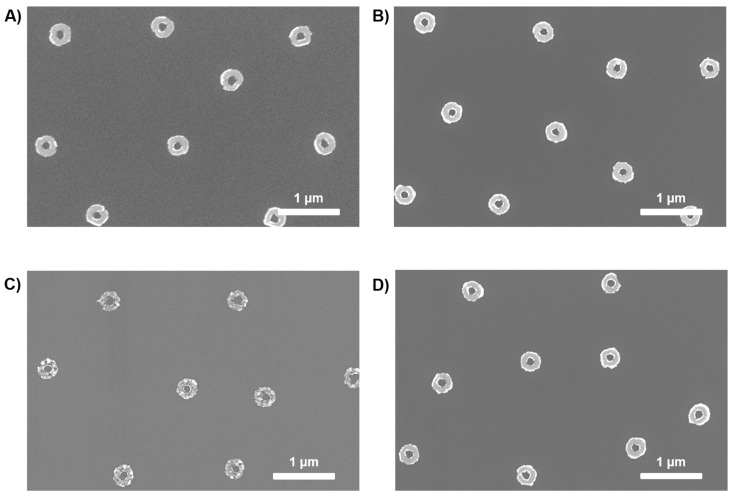
SEM images showing different materials’ 300 nm nanorings fabricated on a different deposition setup: (**A**) Au; (**B**) Pt; (**C**) Ag; (**D**) Cu.

To test the robustness and replicability of the method, the same fabrication route was followed at a different laboratory in another country. This implied completing the entire fabrication route using different equipment and instruments, including distinct experimental conditions of humidity and temperature, to prove that the same route was completed, ensuring the thickness of the resist (75 nm), the PS NP deposition (Figure S7), a 15 nm Ti mask, etching of the PMGI, followed by PS NP tape stripping, and metal deposition. To test further versatility, the method was used to fabricate nanostructures of different materials, including gold (Au), platinum (Pt), silver (Ag), and Cu. As shown in Figure 5, the nanostructures were successfully fabricated, and the presence of non-uniform edges can again be associated with an aggressive lift-off caused by having pillars with non-straight walls.

The reproducibility of these features across the substrate demonstrates the applicability of DC magnetron sputtering in depositing high-fidelity nanoring architectures, even for some metallic materials, which are often considered more reactive and challenging to work with due to oxidation and surface roughening.

This opens the door to a new set of experiments where different setups can be tested to optimize the fabrication of the mentioned nanostructures and try to fabricate nanorings of different materials, including ceramics, that can be relevant in certain industries. That is a great advantage in comparison to the previous method using E-beam PVD, as the number of materials available to be deposited was limited.

### 3.2. Optical Response and Plasmonic Behavior of Cu Nanorings

**Figure 6:**
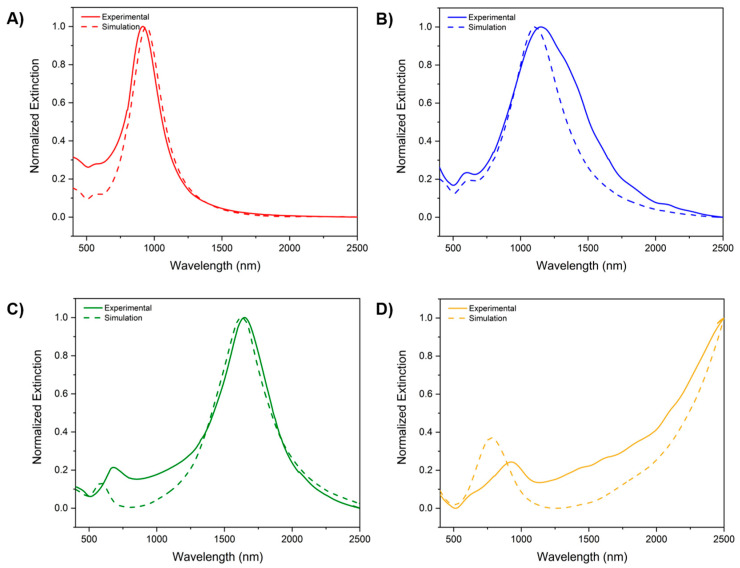
Normalized extinction spectra showing the optical response of the fabricated nanorings vs. simulated results: (**A**) 200 nm (red); (**B**) 300 nm (blue); (**C**) 500 nm (green); (**D**) 800 nm (yellow).

The optical response of the fabricated nanorings was evaluated through extinction spectroscopy and compared with finite-difference time-domain (FDTD) simulations. Figure 6 presents the extinction spectra of nanorings with varying outer diameters (200–800 nm), in both the experimental and simulated contexts.

The extinction spectrum of the 200 nm Cu nanorings (Figure 6A) displays a pronounced LSPR peak centered around 900 nm, with the experimental data closely mirroring the simulated response, with minor blue-shifting. This discrepancy can be attributed to slight deviations in geometrical parameters (e.g., wall thickness and inner diameter) and the presence of surface oxidation, which is not fully accounted for in the simulations; the same can be attributed to the higher energy peak sitting around 600 nm. As the nanoring diameter increases to 300 nm and 500 nm (Figure 6B,C), the LSPR peak shifts progressively toward longer wavelengths (~1200 and 1650 nm, respectively, for the low-energy LSPR peak), entering deeper into the NIR range. Notably, the extinction intensity also increases, indicating enhanced light–matter interaction due to the greater effective interaction volume and increased polarizability of the larger rings. Again, the discrepancies between the experimental and simulated results can be attributed to the presence of oxidation, secondary nanostructures, and some possible aggregations.

For the 800 nm nanorings (Figure 6D), the extinction spectrum reveals a broad and red-shifted response extending beyond 2000 nm, consistent with higher-order plasmon modes and multipolar resonances expected in larger nanostructures. The simulations qualitatively reproduce the spectral features observed experimentally, confirming the power of the computational approach. Nevertheless, variations in ring height, grain structure, and slight asymmetries in the fabricated structures likely contribute to peak broadening and spectral shifts. Furthermore, it is important to mention that the material plays a crucial role in the optical response; our previous work discussed the effect of the material of choice on the optical response [26].

**Figure 7:**
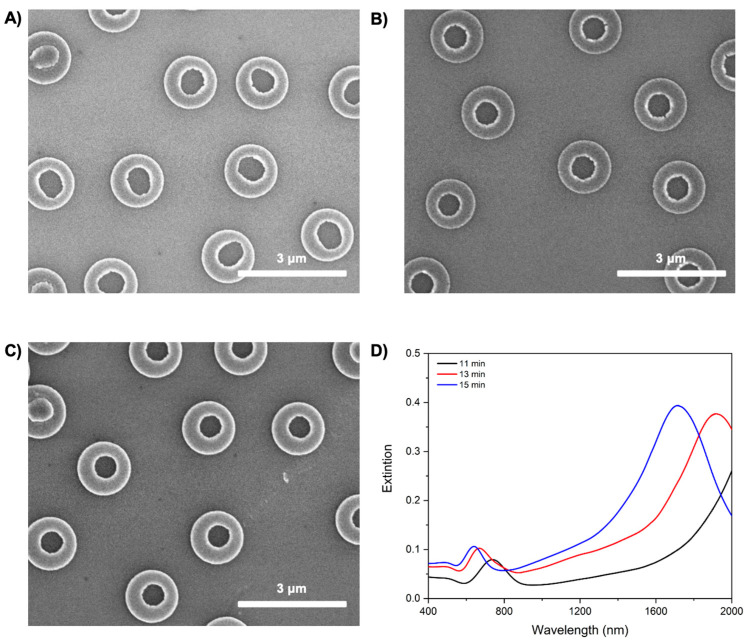
Effect of etching time on 500 nm Cu rings: (**A**) 11 min; (**B**) 13 min; (**C**) 15 min; (**D**) Optical response.

To further investigate the tunability of the Cu nanorings’ optical response, a series of samples with identical nominal diameters (500 nm, for accurate morphological resolution) were subjected to varying etching durations. SEM images in Figure 7A–C show the morphological evolution of the Cu nanorings following etching times of 11, 13, and 15 min, respectively. With increasing etching duration, a progressive thickening of the ring walls is observed, alongside a reduction in the central aperture; all of this as a consequence of thinner PMGI pillars. This evolution is indicative of isotropic etching, which results in decreased metallic volume and altered aspect ratios.

The morphological changes induced by the etching process significantly impact the LSPR characteristics, as illustrated in the extinction spectra shown in Figure 7D. For the shortest etching time (11 min), the extinction spectrum exhibits a prominent LSPR peak. Upon extending the etching to 13 min, the peak shifts toward shorter wavelengths, and the overall intensity seems to increase, suggesting an increasing effective dielectric function due to higher Cu volume. With further etching to 15 min, the extinction maximum continues to blue-shift, and a broader spectral profile is observed.

This trend can be rationalized by considering that the LSPR wavelength is influenced by both the overall size and the wall thickness of the nanorings. The optical response of the nanorings can be described as a hybridization of a superimposed disk and hole [7]. As the wall thickness increases, the energy of the hole and disk becomes more different, leading to less hybridization and a blue shift in the low-energy nanoring resonance. Moreover, a small increase in extinction intensity is consistent with a greater absorption cross-section, reflecting an increased volume of metal with more electrons, contributing to a stronger oscillator dipole.

The observed plasmonic behavior of the Cu nanorings highlights the potential for precise tuning of optical properties through morphological control. The systematic dependence of the LSPR position on both ring diameter and wall thickness affirms the utility of these structures in applications requiring wavelength-specific absorption or scattering, such as NIR-selective filtering and photothermal conversion.

The ability to fabricate Cu nanorings with tailored optical responses using a scalable and relatively low-cost deposition method such as DC magnetron sputtering represents a significant advancement. In contrast to noble metals like Au and Ag, Cu offers economic and material compatibility advantages for integration into large-area thermoplasmonic devices. Although oxidation remains a concern, the reproducibility of the optical features in the extinction spectra suggests that the effect is minimal. However, oxidation can be stabilized or controlled through post-fabrication treatments. There are different literature examples presenting methods for this control of oxidation [41,42].

### 3.3. Thermoplasmonics Response of Cu Nanorings Under Direct Sun Irradiation

**Figure 8:**
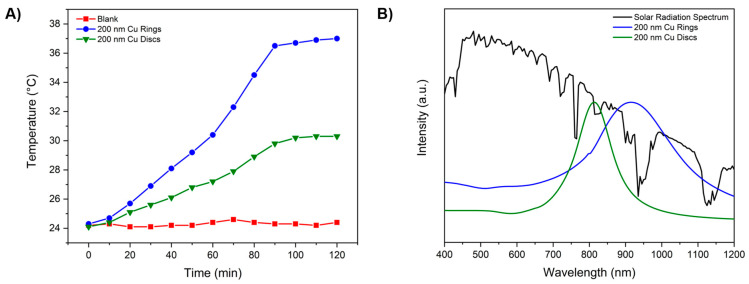
Thermoplasmonic studies: (**A**) evolution of temperature during exposure to solar light for a blank glass (red), a glass substrate containing 200 nm Cu rings (blue), and a glass substrate containing 200 nm Cu disks (green); (**B**) overlap between the solar radiation spectrum (black) and the extinction spectra of 200 nm Cu rings (blue) and 200 nm Cu disks (green).

To study the thermoplasmonic performance of the fabricated 200 nm Cu nanorings (for a better coupling with the solar spectrum), their heating behavior under direct sunlight irradiation was monitored and compared against a blank glass substrate and a glass substrate containing 200 nm Cu nanodisks that we also fabricated by HCL (Figures S8 and S9) [22]. Figure 8A shows the evolution of the surface temperature for the three samples over a period of 2 h. The blank substrate exhibited negligible heating, with its temperature remaining close to ambient conditions throughout the measurement, highlighting that the glass alone does not significantly absorb or convert sunlight into heat within this spectral range.

In contrast, both substrates containing the fabricated nanostructures showed a progressive increase in temperature under sunlight exposure due to the excitation of their LSPR, which effectively transduces incident photons into localized thermal energy. The response of the Cu nanorings was particularly pronounced compared to the nanodisks, reaching a maximum temperature of approximately 37 °C, corresponding to an increase of over 13 °C relative to the ambient temperature. This strong thermoplasmonic activity indicates efficient photon-to-heat conversion, which can be attributed to the unique geometry of the rings. The hollow-core configuration supports hybridized plasmonic modes and facilitates stronger field confinement at the metal–dielectric interface compared to solid disks. Consequently, the nanorings exhibit enhanced absorption cross-sections and more efficient heat generation under broadband sunlight irradiation.

For comparison, the Cu nanodisks demonstrated a moderate heating response, stabilizing at around 30 °C after 100 min of irradiation. While disks also sustain plasmonic resonances, their solid geometry limits the extent of light–matter interaction compared to rings of equivalent dimensions. Moreover, as seen in Figure 8B, the extinction spectrum of Cu disks overlaps to a lower degree with the solar radiation spectrum compared to the one of Cu nanorings; this is due to the broader LSPR peak of the rings. The observed difference between rings and disks thus emphasizes the role of geometry in governing the thermoplasmonic response, something that can be clearly observed by the presence of hybridization modes [7,8,25,26,43]. Consequently, this highlights the potential of nanorings for solar-driven applications. Moreover, higher temperatures can be reached if several types of nanostructures or diverse sizes are employed on the same surface by having a greater coupling with the solar spectrum.

Overall, these findings demonstrate that Cu nanorings fabricated via sputter deposition exhibit superior thermoplasmonic performance under direct sunlight compared to their solid counterparts. Their enhanced heating capability, derived from their distinct geometry, underlines the suitability of nanorings as functional building blocks for solar-responsive devices, where passive control of light and heat is of paramount importance. As mentioned before, oxidation remains a concern, and post-fabrication treatments can be of great interest in order to prevent this oxidation from taking place, allowing a sustained thermoplasmonic activity through time.

## 4. Conclusions

In this work, we present a robust nanofabrication strategy for Cu nanorings by integrating Hole-mask Colloidal Lithography (HCL) with DC magnetron sputtering. The method successfully overcomes the geometrical limitations of angled deposition by introducing a tape-stripping step, which enables the reproducible formation of polymer pillars and ring-shaped apertures across large substrate areas. The optimized protocol proved versatile, yielding nanorings with tunable diameters (200–800 nm) and adaptable to different metallic materials, including Au, Pt, and Ag, thereby highlighting its industrial relevance.

Structural and optical characterization revealed that the hollow-core geometry of the nanorings provides superior plasmonic tunability compared to solid nanodisks. Extinction spectroscopy, supported by numerical simulations, confirmed the dependence of localized surface plasmon resonance (LSPR) on both ring diameter and wall thickness. Moreover, systematic variation in etching parameters allowed for control over morphological features, further expanding the design flexibility of the structures.

The thermoplasmonic performance of Cu nanorings was studied under direct sunlight irradiation, where they exhibited significantly enhanced heating compared to nanodisks. This superior photon-to-heat conversion efficiency arises from their unique ring geometry, which enhances light–matter interaction and absorption cross-sections. Overall, the results establish Cu nanorings fabricated via this route as promising building blocks for solar-responsive applications.

## Data Availability

The original contributions presented in this study are included in the article/Supplementary Material. Further inquiries can be directed to the corresponding author.

## Supplementary Materials

The following supporting information can be downloaded at https://www.mdpi.com/article/10.3390/ma18184371/s1, Supporting figures and tables can be found to complete the research presented. Figure S1: An example of a line scan profilometer measurement of a PMGI thin film on Si substrate, using the spin coating parameters: 60 s, 1000 rpm, 5000 rpm/s. The thickness of the PMGI thin film is determined by the height difference between the bottom of the valley and the surface, in this measured to 74.6 nm; Figure S2: SEM images showing different size nanoparticles distribution on silicon substrates; (A) 200 nm, (B) 300 nm, (C) 500 nm, (D) 800 nm. Magnification was adjusted; Figure S3: SEM image showing a side view of the cross section of a silicon substrate after etching the PMGI underneath the PS NP with O2 plasma; Table S1: Etching times for the different nanorings fabrication according to the size of the used PS NP. Power was fixed to 100W, 50 mTorr, and 100 SCCM O2; Figure S4: SEM images showing the tape striping step; Figure S5: SEM Image after tape stripping 200 nm PS NP; Figure S6: Images of a glass and silicon samples during the fabrication of 500 nm Cu nanorings; Figure S7: SEM images showing different size nanoparticles distribution on silicon substrates in the second experimental site; (A) 200 nm, (B) 300 nm, (C) 500 nm, (D) 800 nm. Magnification was not adjusted in this case; Figure S8: SEM images showing the employed nanostructures: (A) 200 nm Ag rings; (B) 200 nm Ag discs; Figure S9: Optical response for experimental and simulated 200 nm Cu nanodiscs; Figure S10: Diagram of the simulation space setup in Lumerical FDTD for simulating metallic nanorings; (A) Top View, (B) 3D View; Table S2: List of the various simulation space parameters used in the simulation of metallic nanorings using Lumerical FDTD Solutions; Figure S11: Diagram of the physical parameters that characterize the nanoring in the simulation; (A) Top View, (B) 3D View Note that in (B), the nanoring is upside down, so that the light blue is the simulated substrate; Table S3: List of the various simulation parameters specifically for describing the nanorings using Lumerical FDTD Solutions.
