# Supplementary material for: Thermoplasmonic Nanorings via Sputter Deposition
Source: Materials (Basel). 2025 Sep 18;18(18):4371. doi: 10.3390/ma18184371 (PMC12471788; doi:10.3390/ma18184371)
Supplement: Supplementary file 1 [file materials-18-04371-s001.zip › materials-3860914-supplementary.pdf]

Supporting Information

Thermoplasmonic Nanorings via Sputter Deposition

Xavier Baami González [a], Peter K. Petrov [b], Duncan S. Sutherland [a*]

[a] Interdisciplinary Nanoscience Center (iNANO), Aarhus University, Gustav Wieds Vej 14 8000 Aarhus, Denmark

[b] Department of Materials, Imperial College London, Prince Consort Rd 8, South Kensington, London SW7 2AZ, United Kingdom

*Corresponding author:

Duncan S. Sutherland, E-mail: duncan@inano.au.dk

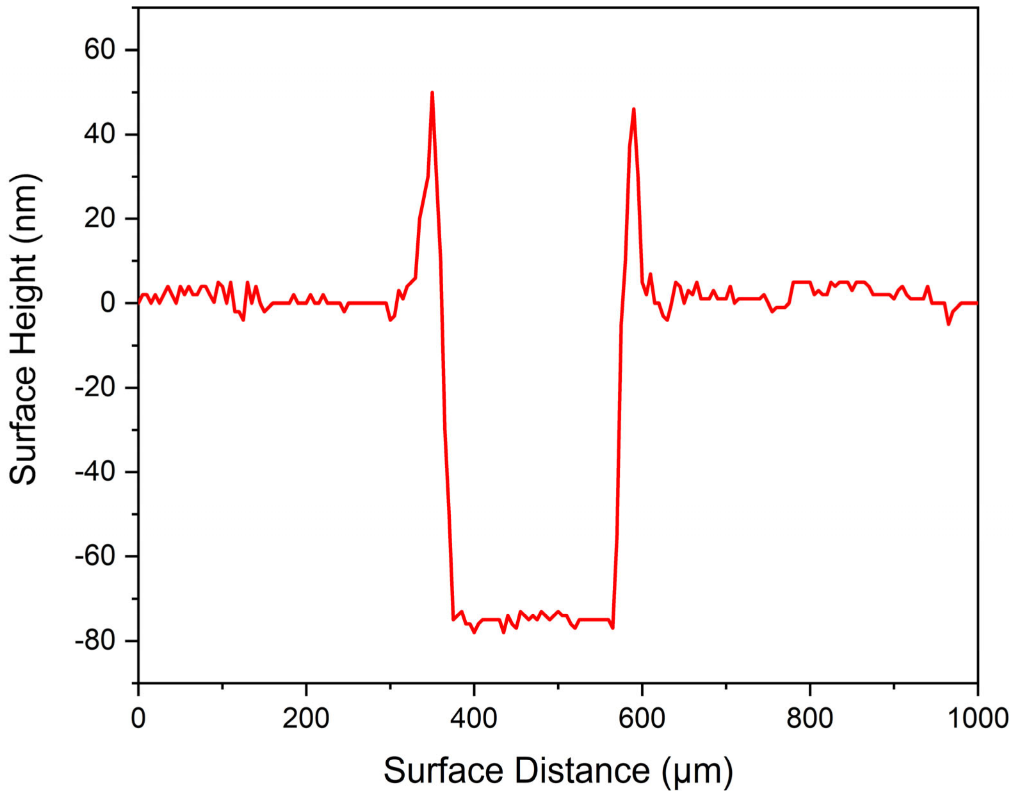

Figure S1: An example of a line scan profilometer measurement of a PMGI thin film on Si substrate, using the spin coating parameters: 60 s, 1000 rpm, 5000 rpm/s. The thickness of the PMGI thin film is determined by the height difference between the bottom of the valley and the surface, in this measured to 74,6 nm.

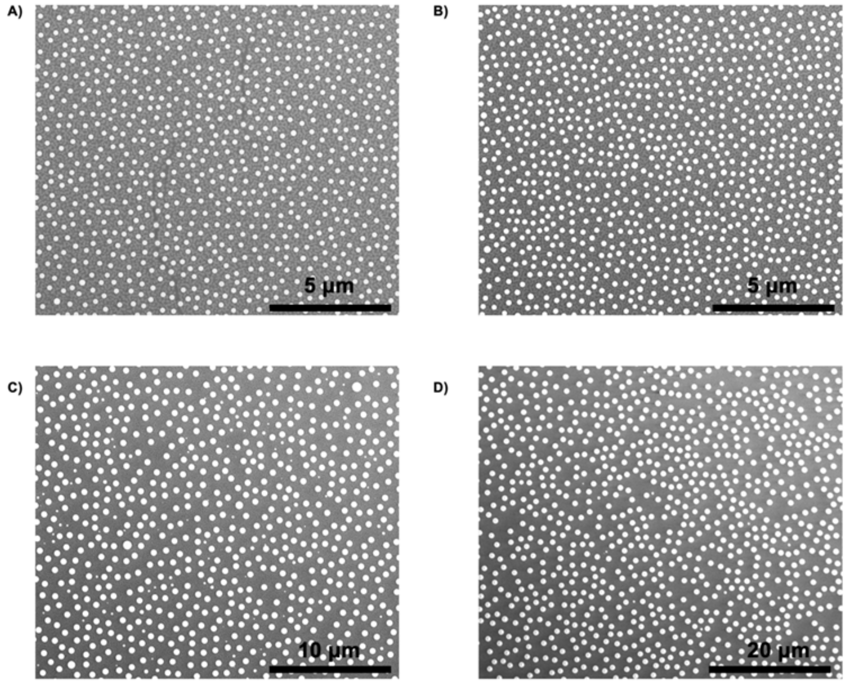

Figure S2: SEM images showing different size nanoparticles distribution on silicon substrates; A) 200nm, B) 300nm, C) 500nm, D) 800nm. Magnification was adjusted.

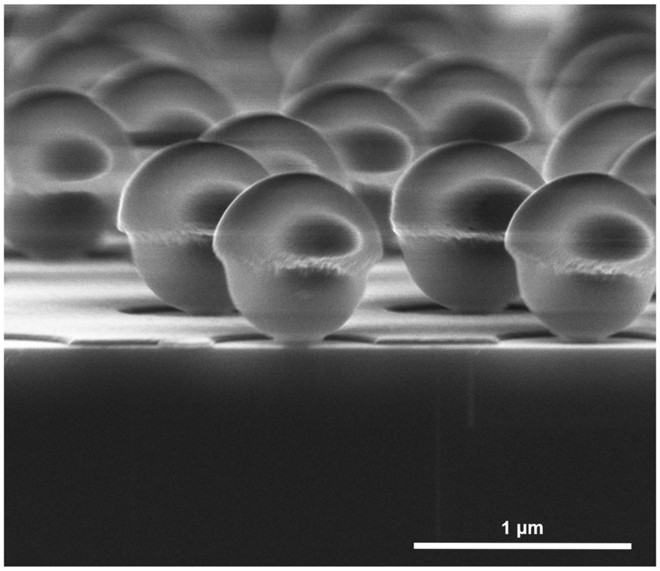

Figure S3: SEM image showing a side view of the cross section of a silicon substrate after etching the PMGI underneath the PS NP with O_2 plasma.

Table S1: Etching times for the different nanorings fabrication according to the size of the used PS NP. Power was fixed to 100W, 50 mTorr, and 100 SCCM O_2.

Particle / Nanoring Size (nm)	Etching time (min)
**200**	**6**
**300**	**8**
**500**	**12**
**800**	**20**

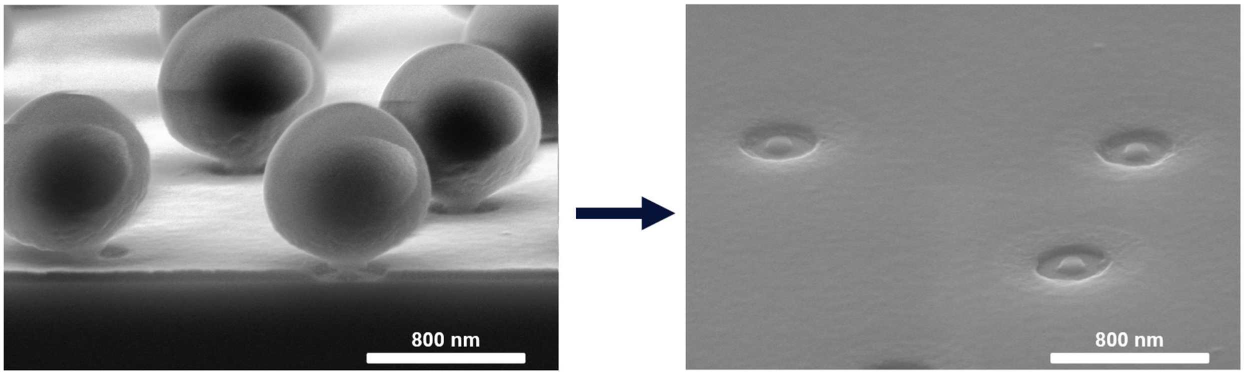

Figure S4: SEM images showing the tape striping step.

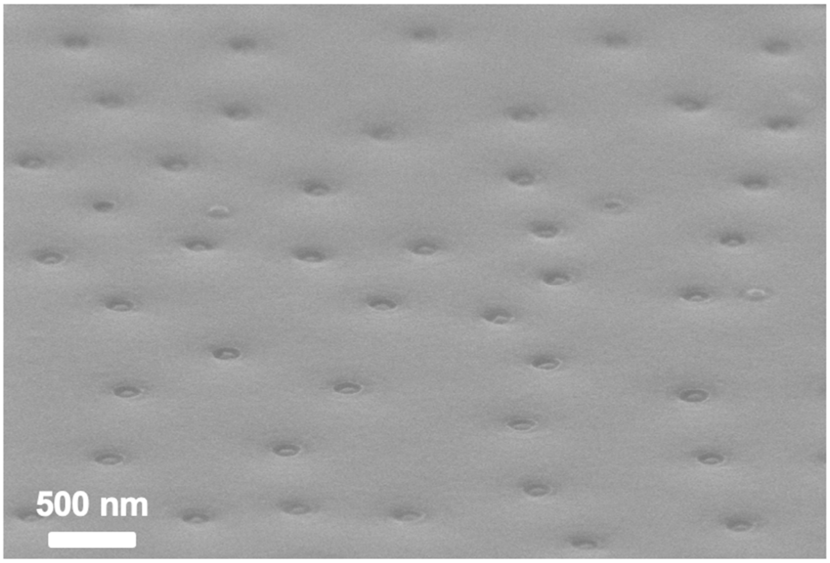

Figure S5: SEM Image after tape stripping 200 nm PS NP.

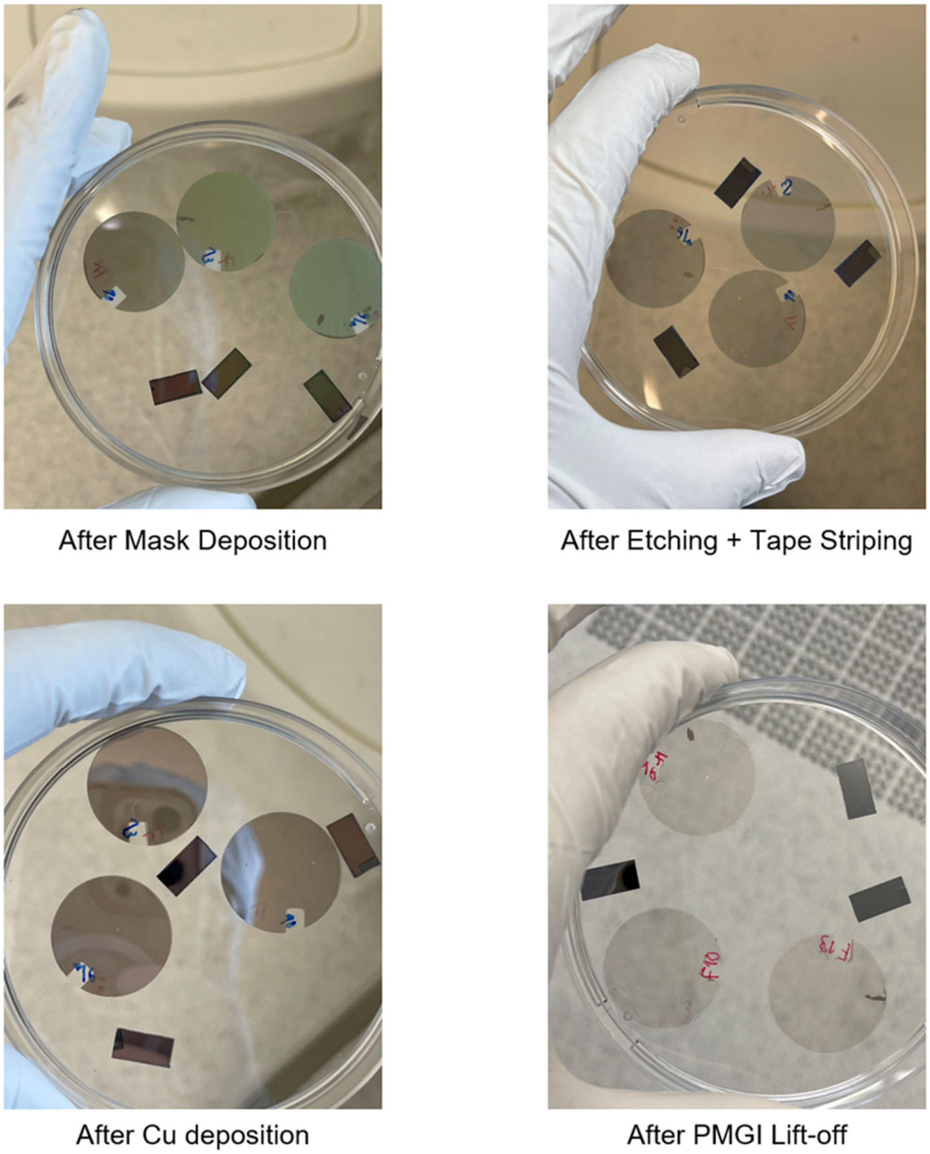

Figure S6: Images of a glass and silicon samples during the fabrication of 500 nm Cu nanorings.

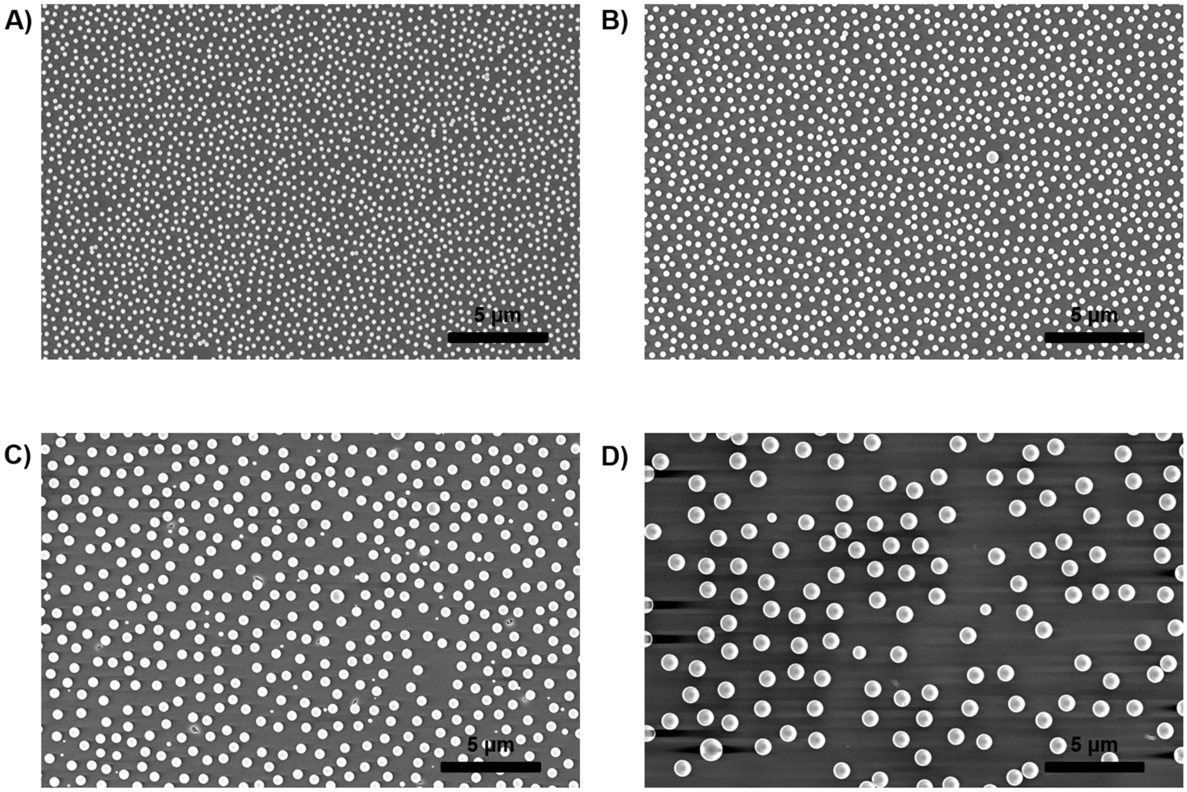

Figure S7: SEM images showing different size nanoparticles distribution on silicon substrates in the second experimental site; A) 200nm, B) 300nm, C) 500nm, D) 800nm. Magnification was not adjusted in this case.

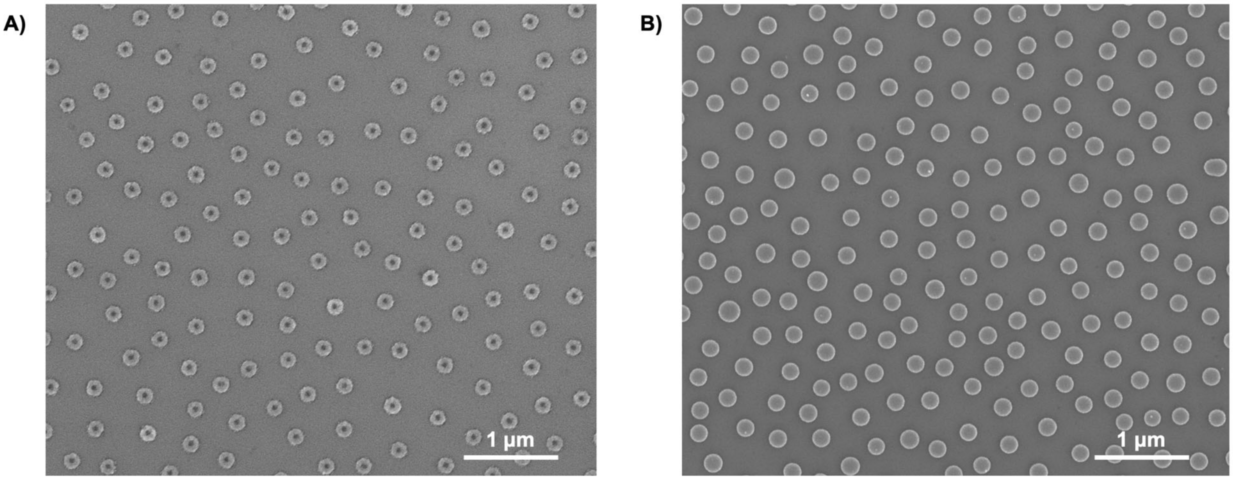

Figure S8: SEM images showing the employed nanostructures: (A) 200 nm Ag rings; (B) 200 nm Ag discs.

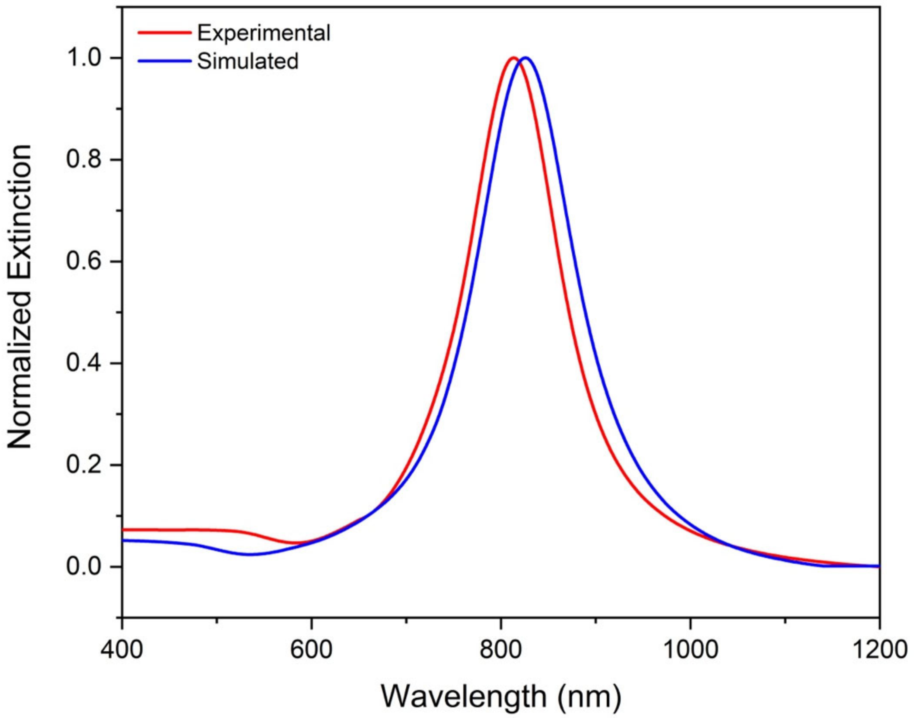

Figure S9: Optical response for experimental and simulated 200 nm Cu nanodiscs.

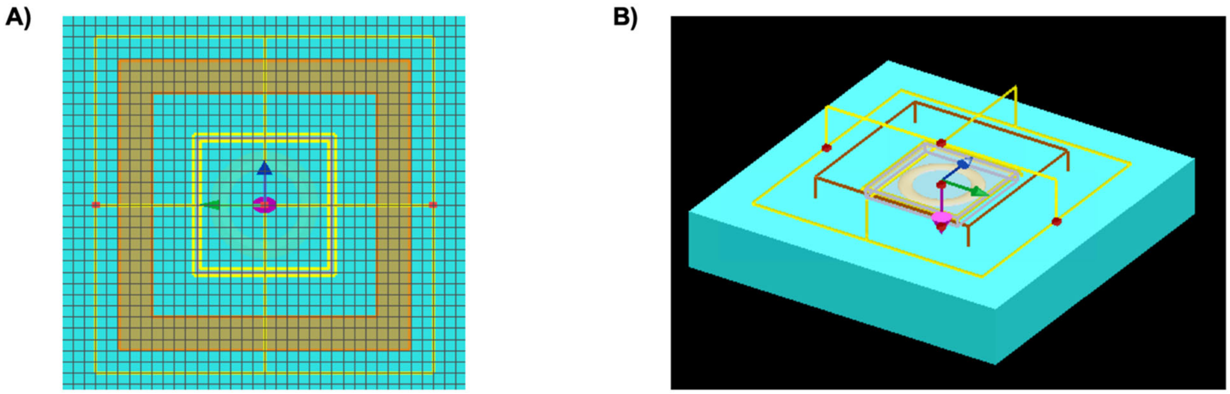

Figure S10: Diagram of the simulation space setup in Lumerical FDTD for simulating metallic nanorings; A) Top View, B) 3D View.

Table S2: List of the various simulation space parameters used in the simulation of metallic nanorings using *Lumerical FDTD Solutions.*

Simulation Space Volume	2000x2000x750 nm
Source Volume	1200x1200x150 nm
Source Wavelength Range	400-2500 nm
Mesh Override Volume	1200x1200x150 nm
Mesh Override Size (Dimensions of Yee cells)	2x2x2 nm
Default Refractive Index of Environment	1
Number of border PML layers	16

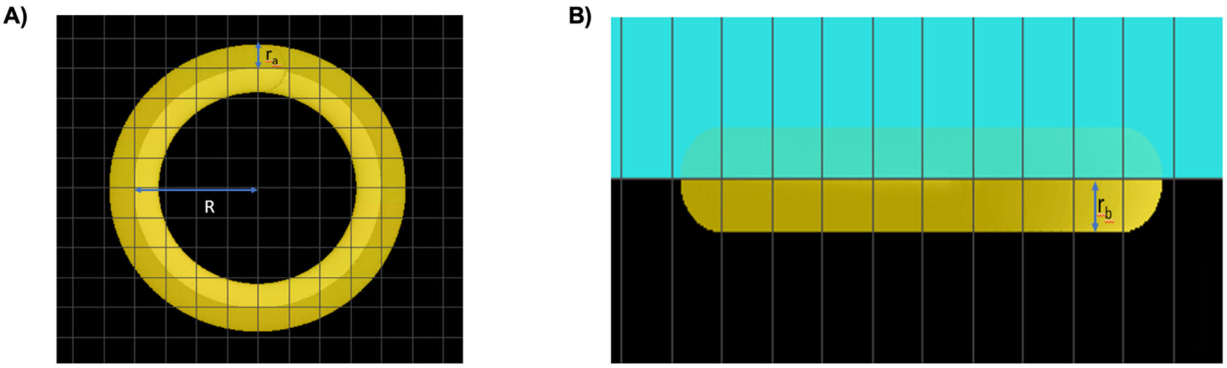

Figure S11: Diagram of the physical parameters that characterize the nanoring in the simulation; A) Top View, B) 3D View Note that in B), the nanoring is upside down, so that the light blue is the simulated substrate.

Table 3: List of the various simulation parameters specifically for describing the nanorings using *Lumerical FDTD Solutions*.

Nanoring size (nm)	R (nm)	r_a (nm)	r_b (nm)
200	65	35	20
300	100	50	20
500	160	90	20
800	290	110	20
